# Test-retest reliability data of functional performance, strength, peak torque and body composition assessments in two different age groups of Kosovan adults

**DOI:** 10.1016/j.dib.2021.106988

**Published:** 2021-03-23

**Authors:** Arben Boshnjaku, Abedin Bahtiri, Kaltrina Feka, Ermira Krasniqi, Harald Tschan, Barbara Wessner

**Affiliations:** a Centre for Sport Science and University Sports, University of Vienna, Auf der Schmelz 6, 1150 Vienna, Austria; b Faculty of Medicine, University of Gjakova ‘Fehmi Agani’, Ismail Qemali, 50000 Gjakova, Kosovo; c Physical Culture Sport and Recreation, Universe College Kosovo, Bardhosh, 10000 Prishtina, Kosovo; d Department of Psychology, Educational Science and Human Movement, University of Palermo, Via Giovanni Pascoli 6, 90144 Palermo, Italy; e Department of Nutritional Sciences, Faculty of Life Sciences, University of Vienna, Althanstraße 14, 1090 Vienna, Austria; f Research Platform Active Ageing, University of Vienna, Althanstraße 14, 1090 Vienna, Austria

**Keywords:** Test-retest reliability, Physical fitness, Body composition, Age-related muscle loss

## Abstract

This article reports test-retest reliability data of laboratory- and field-based performance tests as well as body composition analyses of younger and older Kosovan adults. In total, 57 healthy young (18–35 years) and 61 older (>60 years) participants took part in two identical test sessions, with a median [25^th^ – 75^th^ percentile] of 14 [13–21] days in between. Functional performance tests included 30-s chair stand test (CST), 30-s arm curl test (ACT), six-minutes walking test (6MWT), sit and reach test, timed up and go test (TUG), as well as the assessment of gait speed (GS) at normal and fast pace. Isometric handgrip strength (HGS) was used to estimate strength of the dominant hand. Isokinetic peak torque (PT) and average power (AvgP) for knee extension and flexion were determined at velocities of 60°/s and 120°/s. Body composition assessments included body fat percentage, skeletal muscle mass (SMM) and index (SMI) as well as appendicular skeletal muscle mass (ASMM) and index. Secondary endpoints included self-perceived health status and potential co-morbidities. All performance test outcomes as well as body fat percentage, SMM, ASMM, and self-perceived health were significantly better in young as compared to older participants (*p* < 0.001). Improvements from test to retest were observed for CST (*p* < 0.001), PT_flexion_ (60°/s: *p* = 0.001, 120°/s: *p* = 0.041), AvgP_flexion_ (60°/s: *p* < 0.001, 120°/s: *p* < 0.001), AvgP_extension_ (120°/s: *p* = 0.050), but also for SMM (*p* = 0.021) and SMI (*p* = 0.021). Only for CST and HGS a time x age group interaction was detected (*p* < 0.05). Acceptable reliability (ICC > 0.7) was observed for all parameters in both age groups, except for some of the measures from the isokinetic dynamometry, where ICCs were generally lower in older participants, but fell below 0.7 for AvgP_flexion_ at 60°/s (ICC = 0.6) and at 120°/s (ICC = 0.67) as well as for PT_flexion_ at 120°/s (ICC = 0.69). These data's importance lay upon their potential use in epidemiological studies observing muscle strength, peak torque, power, physical performance and body composition over various age groups, either in the same or similar populations, or for comparison to other populations.

## Specifications Table

**Table:** 

Subject	Sport Sciences, Therapy and Medicine
Specific subject area	Assessment of functional performance, muscle strength, muscle mass, body composition
Type of data	Table
How data were acquired	Stadiometer for height (DT05L, Kinlee, Zhongshan Jinli Electronic Weighing Equipment Co. Ltd, China); Segmental multifrequency bioelectrical impedance analyser for body mass and composition (Inbody 720, Biospace Co., Seoul, Korea); Biodex dynamometer for isokinetic peak torque knee extension and flexion (Biodex System 4 Pro-Isokinetic Dynamometer, Shirley, New York, USA); Handgrip dynamometry for isometric handgrip strength (JAMAR, Patterson Medical, USA); Track (10 m) and stop watch for gait speed (sports hall); Track (3 m), armless chair (46 cm seat height) and stop watch for timed up and go test (sports hall); Armless chair (46 cm seat height) and stop watch for 30-s chair stand test (sport hall); Dumbbell (5 pounds for woman and 8 pounds for men), armless chair (46 cm seat height) and stopwatch for 30-s arm curl test (sports hall); 30-meters shuttle track and stop watch (sports hall) for six-minutes walking test (sports hall); Measuring ruler and armless chair (46 cm seat height) for sit and reach test (sports hall).
Data format	Raw data
Parameters for data collection	Human test-retest study on physical performance and body composition parameters of young (18–35 years) and older (above 60 years) subjects living in the region of Prishtina, Kosovo
Description of data collection	Body composition data were collected in the morning after an overnight fast by the same research team at the Sports Medicine Laboratory at the “Universi College” in Bardhosh, Prishtina. After a break of 30 min and provision of a light standardized meal, isometric, isokinetic and functional performance tests were assessed in the sport hall of the same institution. Retest was performed in the same manner after a period of 19 ± 16 days.
Data source location	Institution: Universi College Kosovo City/Town/Region: Bardhosh, Prishtina, 10,000 Country: Kosovo Latitude and longitude for collected samples/data: 42.7228° and 21.1476°
Data accessibility	Mendeley Data Repository [1]

## Value of the Data

• The data of this article are important for cross-sectional and longitudinal studies observing functional performance, strength, power and body composition assessments throughout the life span, where there is a need to choose tests with similar reliability in younger and older adults.
• Reliability data of laboratory and field tests for functional performance, strength and power as well as body composition allow researchers, clinicians, sport scientists, physiotherapists or other health experts to compare these methods with respect to their measurement accuracy.
• Population-specific data of physical performance in two different age groups and for both sexes are given. Thus, the data can be reused for calculation of population-specific reference ranges needed i.e. for sarcopenia diagnostics in the Kosovan/Balkans area.

## 1 Data Description

The present data focus on test-retest reliability of functional performance tests together with strength, power and body composition assessments in young and older Kosovan adults. The data set comprises various parameters relevant to assess age-related changes in physical performance. Those parameters have been suggested by the European Working Group in Sarcopenia for Older People (EWGSOP) in its initial and revised consensus statements to be used for the diagnosis and treatment of sarcopenia [2,3].

Age group and sex-specific baseline characteristics of the participants including anthropometric data as well as health status are provided Table 1. Test-retest reliability of various functional performance tests included in the senior fitness test manual [4], such as the 30-s chair stand test, 30-s arm curl test, the six-minutes walking test (6MWT), the sit and reach test, the timed up and go test as well as gait speed data are shown in Table 2. In Table 3, test-retest reliability of laboratory-based assessments of isokinetic peak torque as well as average power at two different velocities (60°/s and 120°/s) is shown for knee extension and knee flexion. Finally, Table 4 summarizes test-retest reliability of parameters derived from bioimpedance analyses such as body fat percentage, skeletal muscle mass (SMM), skeletal muscle index (SMI), appendicular skeletal muscle mass (ASMM) and appendicular skeletal muscle index (ASMI).

**Table 1:** Baseline characteristics of participants.

	Young Participants	Young Females	Young Males	Old Participants	Old Females	Old Males	*p-value*
Participants [number (%)]	**57**	26 (45.6%)	31 (54.4%)	**61**	39 (63.9%)	22 (36.1%)	
Age [years]	**22.6** **±** **3.7**	23.6 ± 4.3	21.8 ± 2.9	**70.7** **±** **6.1**	69.6 ± 5.7	72.7 ± 6.5	*<0.001*
Height [m]	**1.75** **±** **0.10**	1.68 ± 0.08	1.81 ± 0.07	**1.64** **±** **7.69**	1.60 ± 5.42	1.70 ± 7.60	*<0.001*
Body mass [kg]	**70.3** **±** **14.0**	62.0 ± 11.4	77.2 ± 12.2	**80.8** **±** **14.7**	79.6 ± 14.0	83.0 ± 15.9	*<0.001*
BMI [kg/m²]	**22.8** **±** **3.3**	22.0 ± 3.4	23.5 ± 3.0	**30.1** **±** **4.9**	30.8 ± 4.6	28.8 ± 5.1	*<0.001*
Body fat [%]	**19.5** **±** **8.6**	23.8 ± 8.5	15.8 ± 6.9	**39.4** **±** **8.2**	43.1 ± 5.3	32.5 ± 8.4	*<0.001*
SMM [kg]	**34.6** **±** **7.8**	25.6 ± 5.8	36.7 ± 5.2	**26.5** **±** **4.9**	24.2 ± 3.4	30.6 ± 4.5	*<0.001*
SMI [kg/m²]	**10.2** **±** **1.6**	9.0 ± 1.4	11.2 ± 1.0	**9.8** **±** **1.1**	9.4 ± 0.9	10.6 ± 1.0	*0.075*
ASMM [kg]	**23.9** **±** **5.9**	19.3 ± 4.3	27.7 ± 4.1	**20.3** **±** **3.9**	18.6 ± 2.9	23.5 ± 3.6	*<0.001*
ASMI [kg/m²]	**7.7** **±** **1.2**	6.8 ± 1.0	8.4 ± 0.7	**7.5** **±** **0.9**	7.2 ± 0.9	8.1 ± 0.8	*0.398*
Self-perception of health [good/not good], n (%)	**47/10** **(82.5/17.5)**	23/3 (88.5/11.5)	24/7 (77.4/22.6)	**27/34** **(44.3/55.7)**	19/20 (48.7/51.3)	8/14 (36.4/63.6)	*<0.001*
Chronic diseases, n (%)	**0 (0)**	0 (0)	0 (0)	**46 (75.4)**	29 (74.4)	17 (77.3)	*<0.001*
Cardiovascular disease, n (%)	**0 (0)**	0 (0)	0 (0)	**28 (45.9)**	19 (48.7)	9 (40.9)	*<0.001*
Diabetes, n (%)	**0 (0)**	0 (0)	0 (0)	**11 (18.0)**	9 (23.1)	2 (9.1)	*0.001*
Osteoporosis, n (%)	**0 (0)**	0 (0)	0 (0)	**7 (11.5)**	5 (12.8)	2 (9.1)	*0.008*
Degenerative rheumatic problems, n (%)	**0 (0)**	0 (0)	0 (0)	**11 (18.0)**	6 (15.4)	5 (22.7)	*0.003*

*Abbreviations:* BMI (Body Mass Index); SMM (Skeletal Muscle Mass); SMI (Skeletal Muscle Index); ASMM (Appendicular Skeletal Muscle Mass); ASMI (Appendicular Skeletal Muscle Index). Values are shown as mean ± standard deviation; p-values refer to differences between young and old participants (independent student's *t*-test for continuous variables and Chi-square test for categorical variables).

**Table 2:** Test-retest reliability of functional performance tests and isometric handgrip strength.

Functional performance test	Age group (years)	Test (Mean ± SD)	Retest (Mean ± SD)	Difference (Mean ± SD)	95% CI of Difference	Typical error (95% CI)	ICC (95% CI)	Time	Age group	Sex	Time × Age group	Time × Sex
30-s chair stand [rep]	18–35	23.8 ± 5.9	24.2 ± 5.7	0.5 ± 1.9	−0.05 → 0.96	1.34 (1.13–1.65)	0.95 (0.91–0.97)	**<0.001**	**<0.001**	**<0.001**	**0.007**	0.838
	60 +	11.7 ± 3.3	13.1 ± 3.0	**1.4** **±** **1.6^⁎⁎⁎^**	0.95 → 1.77	1.13 (0.96–1.37)	0.80 (0.37–0.92)					
30-s arm curl [rep]	18–35	27.7 ± 6.6	27.8 ± 6.5	0.1 ± 2.3	−0.53 → 0.67	1.60 (1.35–1.96)	0.94 (0.90–0.97)	0.065	**<0.001**	**<0.001**	0.157	0.628
	60 +	14.7 ± 2.7	15.3 ± 3.4	**0.6** **±** **2.1^⁎⁎^**	0.02 → 1.10	1.49 (1.27–1.82)	0.76 (0.62–0.85)					
6MWT [m]	18–35	748.3 ± 102.7	750.3 ± 105.8	2.0 ± 27.1	−5.16 → 7.20	19.19 (16.20–23.54)	0.97 (0.94–0.98)	0.355	**<0.001**	**<0.001**	0.654	0.813
	60 +	425.1 ± 143.8	431.5 ± 142.1	6.4 ± 43.2	−4.66 → 17.44	30.51 (25.90–37.15)	0.95 (0.93–0.97)					
Sit and reach [cm]	18–35	5.5 ± 7.1	5.6 ± 6.8	0.1 ± 1.3	−0.23 → 0.44	0.89 (0.75–1.09)	0.98 (0.97–0.99)	0.267	**<0.001**	0.107	0.581	0.879
	60 +	−1.3 ± 9.4	−1.0 ± 8.8	0.3 ± 2.7	−0.40 → 0.97	1.90 (1.61–2.31)	0.96 (0.93–0.97)					
Timed up and go [s]	18–35	4.1 ± 0.6	4.0 ± 0.6	−0.1 ± 0.3	−0.14 → 0.04	0.24 (0.20–0.29)	0.84 (0.74–0.90)	0.806	**<0.001**	**<0.001**	0.520	0.070
	60 +	7.2 ± 2.1	7.3 ± 2.4	0.1 ± 0.8	−0.12 → 0.30	0.58 (0.49–0.70)	0.94 (0.89–0.96)					
Gait speed [m/s]	18–35	1.4 ± 0.2	1.4 ± 0.2	0.0 ± 0.1	−0.01 → 0.03	0.05 (0.05–0.07)	0.89 (0.82–0.93)	0.595	**<0.001**	**0.045**	0.347	0.869
	60 +	1.1 ± 0.2	1.1 ± 0.2	0.0 ± 0.1	−0.03 → 0.02	0.07 (0.06–0.08)	0.91 (0.85–0.94)					
Gait speed - fast [m/s]	18–35	2.1 ± 0.3	2.1 ± 0.3	0.0 ± 0.2	−0.07 → 0.06	0.18 (0.15–0.21)	0.84 (0.74–0.90)	0.255	**<0.001**	**<0.001**	0.383	0.854
	60 +	1.5 ± 0.3	1.5 ± 0.3	0.0 ± 0.1	−0.07 → 0.00	0.10 (0.09–0.12)	0.91 (0.86–0.95)					
Handgrip strength [kg]	18–35	41.5 ± 10.7	42.3 ± 11.0	**0.8** **±** 2.7*	0.10 → 1.52	1.88 (1.59–2.31)	0.97 (0.94–0.98)	0.339	**<0.001**	**<0.001**	**0.034**	0.229
	60 +	29.2 ± 9.2	28.8 ± 9.3	−0.4 ± 2.7	−1.07 → 0.31	1.90 (1.61–2.31)	0.96 (0.93–0.97)					

*Abbreviations:* 6MWT (Six-minutes walking test); rep (repetitions); SD (standard deviation); CI (confidence interval); ICC (interclass correlation coefficient); *** *p*<0.001, ** *p*<0.01, * *p*<0.05 between test and retest as analysed per paired *t*-test; main effects of time, age group and sex as well as their interaction with time were determined by three-way mixed ANOVA, exact p-values are shown;; significant differences are marked in bold; young participants (*n* = 57), older participants (*n* = 61).

**Table 3:** Test-retest reliability of peak torque and average power assessments for knee extension and flexion.

	Age group (years)	Test (Mean ± SD)	Retest (Mean ± SD)	Difference (Mean ± SD)	95% CI of Difference	Typical Error (95% CI)	ICC (95% CI)	Time	Age group	Sex	Time × Age group	Time × Sex
Peak torque extension, 60°/s [Nm]	18–35	153.7 ± 60.2	154.8 ± 52.2	1.1 ± 30.1	−6.86 → 9.10	21.26 (17.95–26.08)	0.86 (0.77–0.92)	0.296	**<0.001**	**<0.001**	0.633	0.691
	60+	68.5 ± 36.7	70.9 ± 42.8	2.5 ± 25.1	−4.12 → 9.06	17.71 (14.98–21.69)	0.80 (0.69–0.88)					
Peak torque flexion, 60°/s [Nm]	18–35	94.9 ± 43.2	102.8 ± 44.4	**7.8** **±** **19.9^⁎⁎^**	2.53 → 13.11	14.09 (11.89–17.28)	0.88 (0.79–0.93)	**0.001**	**<0.001**	**<0.001**	0.451	0.284
	60+	44.2 ± 21.6	48.9 ± 24.6	**4.7** **±** 17.6*	0.03 → 9.27	12.41 (10.49–15.20)	0.70 (0.54–0.81)					
Avg power extension, 60°/s [W]	18–35	83.2 ± 36.1	84.3 ± 32.9	1.1 ± 19.9	−4.18 → 6.35	14.03 (11.85–17.22)	0.84 (0.74–0.90)	0.151	**<0.001**	**<0.001**	0.470	0.438
	60+	30.7 ± 16.6	33.4 ± 21.4	2.7 ± 14.3	−1.06 → 6.45	10.10 (8.54–12.37)	0.72 (0.57–0.82)					
Avg power flexion, 60°/s [W]	18–35	56.7 ± 29.2	63.2 ± 31.9	**6.5** **±** **14.3^⁎⁎^**	2.69 → 10.30	10.14 (8.56–12.44)	0.87 (0.76–0.93)	**<0.001**	**<0.001**	**<0.001**	0.255	**0.007**
	60+	21.9 ± 11.3	24.8 ± 13.7	**2.9** **±** 10.6*	0.11 → 5.69	7.52 (6.35–9.20)	0.63 (0.44–0.62)					
Peak torque extension, 120°/s [Nm]	18–35	115.9 ± 48.5	116.5 ± 44.5	0.6 ± 22.3	−5.31 → 6.55	15.80 (13.34–19.38)	0.89 (0.81–0.93)	0.202	**<0.001**	**<0.001**	0.379	0.825
	60+	46.4 ± 27.6	50.0 ± 30.8	3.5 ± 17.4	−1.03 → 8.10	12.28 (10.38–15.03)	0.82 (0.71–0.89)					
Peak torque flexion, 120°/s [Nm]	18–35	80.8 ± 42.5	85.0 ± 40.8	4.2 ± 29.2	−3.53 → 11.96	20.64 (17.42–25.32)	0.75 (0.62–0.85)	**0.041**	**<0.001**	**<0.001**	0.889	0.942
	60+	33.6 ± 18.3	38.5 ± 18.8	**4.9** **±** 14.1*	1.18 → 8.61	9.99 (8.45–12.23)	0.69 (0.51–0.81)					
Avg power extension 120°/s [W]	18–35	115.4 ± 56.5	118.3 ± 55.5	2.9 ± 31.0	−5.34 → 11.13	21.94 (18.53–26.92)	0.85 (0.75–0.91)	**0.050**	**<0.001**	**<0.001**	0.449	0.553
	60+	36.3 ± 24.9	42.0 ± 31.4	**5.7** **±** 19.4*	0.63 → 10.83	13.72 (11.60–16.79)	0.75 (0.61–0.85)					
Avg power flexion 120°/s [W]	18–35	84.5 ± 48.8	94.8 ± 52.4	**10.3** **±** **24.0^⁎⁎^**	3.96 → 16.67	16.93 (14.30–20.77)	0.87 (0.76–0.93)	**<0.001**	**<0.001**	**<0.001**	0.327	0.116
	60+	27.9 ± 18.9	33.7 ± 21.7	**5.7** **±** **16.0^⁎⁎^**	1.52 → 9.93	11.31 (9.56–13.84)	0.67 (0.48–0.79)					

*Abbreviations:* Avg (average); SD (standard deviation); CI (confidence interval); ICC (interclass correlation coefficient); ** *p*<0.01, * *p*<0.05 between test and retest as analysed per paired *t*-test; significant differences are marked in bold; main effects of time, age group and sex as well as their interactions were determined by three-way mixed ANOVA and exact p-values are shown; young participants (*n* = 57), older participants (*n* = 58).

**Table 4:** Test-retest reliability of body composition parameters.

	Age group (years)	Test (Mean ± SD)	Retest (Mean ± SD)	Difference (Mean ± SD)	95% CI of Difference	Typical Error (95% CI)	ICC (95% CI)	Time	Age group	Sex	Time x Age group	Time x sex
Body fat (%)	18–35	19.5 ± 8.6	19.5 ± 8.3	0.0 ± 1.4	−0.38 → 0.37	1.00 (0.84–1.32)	0.99 (0.98–0.99)	0.595	**<0.001**	**<0.001**	0.584	0.148
	60 +	39.4 ± 8.2	39.1 ± 8.1	−0.3 ± 1.6	−0.67 → 0.15	1.10 (0.93–1.35)	0.98 (0.97–0.99)					
Skeletal muscle mass (kg)	18–35	31.8 ± 7.6	32.0 ± 7.6	0.2 ± 0.7	−0.03 → 0.36	0.52 (0.44–0.64)	1.00 (0.99–1.00)	**0.021**	**<0.001**	**<0.001**	0.851	0.411
	60 +	26.5 ± 4.9	26.7 ± 4.7	**0.2** **±** 0.8*	0.02 → 0.45	0.59 (0.50–0.72)	0.98 (0.97–0.99)					
Skeletal muscle index (kg/m^2^)	18–35	10.2 ± 1.5	10.3 ± 1.5	0.1 ± 0.2	−0.01 → 0.11	0.17 (0.14–0.20)	0.99 (0.98–0.99)	**0.013**	0.420	**<0.001**	0.579	0.327
	60 +	9.8 ± 1.1	9.9 ± 1.1	**0.1** **±** 0.3*	0.02 → 0.18	0.23 (0.19–0.28)	0.95 (0.92–0.97)					
Appendicular skeletal muscle mass (kg)	18–35	23.9 ± 5.9	23.9 ± 5.9	0.0 ± 0.5	−0.10 → 0.17	0.36 (0.30–0.44)	1.00 (0.99–1.00)	0.802	**0.001**	**<0.001**	0.960	0.816
	60 +	20.3 ± 3.9	20.3 ± 3.9	0.0 ± 1.5	−0.35 → 0.43	1.07 (0.91–1.31)	0.93 (0.88–0.96)					
Appendicular skeletal muscle index (kg/m^2^)	Young	7.7 ± 1.2	7.7 ± 1.2	0.0 ± 0.2	−0.03 → 0.06	0.12 (0.10–0.14)	0.99 (0.98–0.99)	0.813	0.782	**<0.001**	0.968	0.865
	60 +	7.5 ± 0.9	7.5 ± 0.9	0.0 ± 0.6	−0.14 → 0.17	0.42 (0.35–0.51)	0.80 (0.69–0.88)					

*Abbreviations:* SD (standard deviation); CI (confidence interval); ICC (interclass correlation coefficient); * *p*<0.05 between test and retest as analysed per paired *t*-test; main effects of time, age group and sex as well as their interaction with time were determined by three-way ANOVA, exact p-values are shown, significant differences are marked in bold; young participants (*n* = 57), older participants (*n* = 60).

## 2 Experimental Design, Materials and Methods

### 2.1 Experimental design and subjects

Young participants were recruited via advertisements in the city of Prishtina (Kosovo). Older subjects were approached via the Kosovo Pensioners’ Association. In total, 57 healthy younger (18–35 years) and 61 older (>60 years) participants took part in the study. Two health professionals evaluated self-perceived health status and co-morbidities by adhering to the WHO-STEPS instrument [5]. Inclusion criteria involved both sexes, an age between 18 and 35 or above 60 years, living in the region of Prishtina, Kosovo and lack of any underlying health condition that would prevent the conduction of the physical performance tests.

Measurements took place from August 2016 until January 2017, in the Sports Medicine Laboratory and the sports hall at the “Universi College” in Prishtina, Kosovo. For assessing test-retest reliability of physical performance, strength and power parameters, participants were asked to take part in two identical test sessions. Study participants were instructed to repeat the test within 1 to 16 weeks, with a strong encouragement to finalize the second test within one to four weeks. This was achieved by 110 (93.2%) persons finally leading to a period of 7–97 days in between the two tests (median [25th–75th percentile]: 14 [13–21] days).

All data were collected in the morning after an overnight fast, uniformly by the same research team consisting of three qualified persons, with the same person being responsible for a specific test. Data collection started at the laboratory with reporting the individual's personal information, anthropometric data and body composition. After a break of 30 min during which a light standardized meal was provided, isokinetic peak torque, handgrip strength and functional performance were assessed in the laboratory or the sport hall of the same institution.

### 2.2 Anthropometric and body composition

Anthropometric data collection followed standardized procedures by the International Standards for Anthropometric Assessment [6] with participants wearing light indoor clothing and being barefoot. Height was measured by means of a portable stadiometer with a precision of 5 mm using the stretch stature method (DT05L, Kinlee, Zhongshan Jinli Electronic Weighing Equipment Co. Ltd, China). Body mass and composition were measured on a segmental multifrequency bioelectrical impedance analysis device with operating frequencies of 1, 5, 50, 250, 500 kHz and 1 mHz (Inbody 770 device, Biospace Co., Ltd., Seoul, Korea). Participants were instructed to stand upright with the soles of both feet over the tactile foot electrodes while holding the hand electrodes. Data output (as determined using the manufacturer's algorithm) included body fat and skeletal muscle mass (SMM) and appendicular skeletal muscle mass (ASMM) being the sum of the lean mass of both arms and legs. Body mass index (BMI), skeletal muscle index (SMI) and appendicular skeletal muscle index (ASMI) were determined as body mass, SMM or ASMM divided by height squared (expressed as kg/m^2^) [7].

### 2.3 Muscle strength and power assessments

Isometric handgrip strength of the self-reported dominant hand was measured by portable hydraulic handgrip dynamometry (JAMAR, Patterson Medical, USA). Upon a brief demonstration and adjustment for hand-size, participants were asked to squeeze the dynamometer for a maximal isometric contraction time of 4–5 s. This was performed on two different trials with one minute rest in between and the better results being considered for analyses [8].

Isokinetic concentric peak torque and average power of knee extensors and flexors were measured in a sitting position at 60°/s and 120°/s on the self-reported dominant leg (Biodex System 4 Pro-Isokinetic Dynamometer, Shirley, New York, USA). The testing range of motion was set from 10–90° of knee flexion. Subjects were instructed to use the support handles at the side of the machine while shin, thigh, pelvic, and upper crossing torso stabilization straps were used for fixation [9].

### 2.4 Functional performance measurements

To assess lower body strength (endurance), the 30-s chair-stand test was used [4]. Participants were instructed to stand up and sit down with arms crossed over the chest as often as possible within 30 s from a 46 cm high armless chair that was placed against a wall. The test administrator stood next to the participant, measured the time by using a stop watch, while counting the number of full stands completed in 30 s between the start and the stop sign that he signalled. The very last attempt was counted, if the subject mastered more than 50% of the range of motion before the timeline.

Upper body strength (endurance) was assessed by the 30-s arm curl test [4], with the participant lifting a dumbbell (five pounds for woman and eight pounds for men) on the self-reported dominant side, while sitting on an armless chair. Similarly to the 30-seconds chair-stand test, the tester stood next, holding and controlling time with a stop watch and counting the number of biceps curls (elbow flexion/extension with supination) completed in 30 s. Again, the very last attempt was counted, if the subject mastered more than 50% of the range of motion before the timeline.

The six minutes walking test (6MWT) was used to assess aerobic endurance [4,10], with participants being instructed to walk as fast as possible on a 30-m shuttle track for six minutes. While recording the time, tester signalled the starting point, remaining time after every minute (5, 4, 3, 2, 1 min to go), the last 15 s reminder and the stopping sign. Participants were allowed to reduce the speed or even to rest, if the speed was too high to be sustained. The covered distance in meters upon the completion of 6MWT was registered as the test result.

To assess lower body flexibility, the chair sit and reach test was used [4]. Participants were sitting on the front edge of a 46 cm high chair placed against a wall. The dominant leg was extended to the front, with the heel on the floor and the foot dorsiflexed at about 90^°^. The other foot was bent and stabilised on the floor with the sole of the foot. Participants were asked to slowly bend forward at the hip joint while keeping the spine and head as straight as possible, reaching down to the toes above the extended leg with palms down on top of each other and the middle fingers tips even. This position was held for a brief 2-s static position, whereas the test administrator registered the reached distance using a ruler parallel to the lower leg. Reaches short of the toes were registered as minus scores, whereas those beyond the toes were registered as plus scores.

Timed up and go (TUG) test served to assess mobility [4,11], with participants being instructed to stand up from an armless chair, walk a distance of three meters, turn around a mark, walk back to the chair and sit down. The test administrator signalled the start of the test and registered the time using a stopwatch upon the completion of the task.

Gait speed was assessed at normal and fast speed [10]. Participants were instructed to walk at a “normal, comfortable speed” (gait speed) and “as fast as you can safely walk” (gait speed fast) in two consecutive trials on a marked 10-m path, with the first two meters used for acceleration and the two last meters for deceleration. Upon the starting signal, time for completion of the middle six meters was measured by using a stopwatch. The better result of the two trials was used and expressed in m/s [8]. Two older subjects (3.3%) had to use a cane to perform the tasks that involve walking (6MWT, TUG and gait speed).

### 2.5 Statistical analyses

Statistical evaluations were performed using IBM SPSS Statistics for Windows Version 27.0 (IBM Corp, Armonk, NY). Descriptive statistics including mean and standard deviation for continuous variables and relative frequencies for categorical variables are provided. Differences between age groups were calculated using independent *t*-test for continual variables and χ2 test for categorical variables. Test-retest reliability was determined by using respective spread sheets as provided by Hopkins [12]. Differences between test and retest data were described by mean differences including standard deviation and 95% confidence interval (95% CI). Furthermore, the typical error (95%CI) was assessed.

Intraclass correlation coefficients (ICC) and their 95% CI were calculated IBM Statistics version 27 based on a single-rating, absolute agreement, two-way mixed effects model [13,14]. Based on Koo and Li [14], an ICC between 0.50 and 0.90 would be considered as ‘moderate’ or ‘good’. With a sample size of *n* = 61 per group we would be able to ensure that for an ICC of 0.80 the lower limit of a 95% one-sided confidence limit is not less than 0.65 with an 80% assurance probability [15].

Finally, a three-way mixed ANOVA was performed providing data for main effects of time, age group and sex as well as time × sex and time × age group interactions to assess whether general test-retest results would be affected by age group or sex.

## Ethics Statement

The work described has been carried out in accordance with The Code of Ethics of the World Medical Association [16] for experiments involving humans, and was approved by the Ethical-Professional Committee of the University Clinical Centre of Kosovo (no 1246/15.09.2016). Participation was voluntary, with written informed consent obtained from all participants.

## CRediT Author Statement

**Arben Boshnjaku:** Conceptualization, Methdodology, Investigation, Data curation, Writing - Original draft preparation; **Abedin Bahtiri**: Investigation, Reviewing final manuscript; **Kaltrina Feka**: Investigation, Reviewing final manuscript; **Ermira Krasniqi**: Investigation, Reviewing final manuscript; **Harald Tschan:** Methodology, Writing- Reviewing and Editing; **Barbara Wessner:** Conceptualization, Supervision, Data curation, Writing – Reviewing and Editing.

## Declaration of Competing Interest

The authors declare that they have no known competing financial interests or personal relationships, which have or could be perceived to have influenced the work reported in this article.
